# Balancing Conservation with National Development: A Socio-Economic Case Study of the Alternatives to the Serengeti Road

**DOI:** 10.1371/journal.pone.0130577

**Published:** 2015-07-22

**Authors:** J. Grant C. Hopcraft, Gerald Bigurube, James Daudi Lembeli, Markus Borner

**Affiliations:** 1 Boyd Orr Centre for Population and Ecosystem Health; Institute of Biodiversity, Animal Health and Comparative Medicine, University of Glasgow, Glasgow, G12 8QQ, United Kingdom; 2 Frankfurt Zoological Society, Box 14935, Arusha, Tanzania; 3 Parliamentary Committee on Land, Natural Resources and Environment, Tanzania, P.O. Box 1065, Kahama, Tanzania; University of Washington, UNITED STATES

## Abstract

Developing countries often have rich natural resources but poor infrastructure to capitalize on them, which leads to significant challenges in terms of balancing poverty alleviation with conservation. The underlying premise in development strategies is to increase the socio-economic welfare of the people while simultaneously ensuring environmental sustainability, however these objectives are often in direct conflict. National progress is dependent on developing infrastructure such as effective transportation networks, however roads can be ecologically catastrophic in terms of disrupting habitat connectivity and facilitating illegal activity. How can national development and conservation be balanced? The proposed Serengeti road epitomizes the conflict between poverty alleviation on one hand, and the conservation of a critical ecosystem on the other. We use the Serengeti as an exemplar case-study in which the relative economic and social benefits of a road can be assessed against the ecological impacts. Specifically, we compare three possible transportation routes and ask which route maximizes the socio-economic returns for the people while minimizing the ecological costs. The findings suggest that one route in particular that circumnavigates the Serengeti links the greatest number of small and medium sized entrepreneurial businesses to the largest labour force in the region. Furthermore, this route connects the most children to schools, provisions the greatest access to hospitals, and opens the most fertile crop and livestock production areas, and does not compromise the ecology and tourism revenue of the Serengeti. This route would improve Tanzania’s food security and self-reliance and would facilitate future infrastructure development which would not be possible if the road were to pass through the Serengeti. This case study provides a compelling example of how a detailed spatial analysis can balance the national objectives of poverty alleviation while maintaining ecological integrity.

## Introduction

Reducing poverty is a dominant theme in sustainable development and the governments of developing countries are often faced with the problem of how to spend limited amounts of capital in the most effective way and without compromising their natural resources. Indeed, poverty alleviation is a central mandate for international lending institutions such as the World Bank, the International Monetary Fund, the European Commission, and bilateral funding agencies that recognize the crucial role of integrating people into the economic, social and political life of the nation [1]. However, despite these common aims there remains much uncertainty as to the most effective approach to alleviate poverty especially given the risks of long-term national debt, aid dependency, and ecologically unsustainable practices.

The transition of countries from “developing” to “newly industrialized” is generally marked by several common features that result from optimizing parallel objectives. These features are: strong agricultural economies with growing industrial sectors (in particular manufacturing fuelled by a large well-connected labour force), an open-market economy with access to international trade especially with neighbours, strong capital investment, and rapid growth of urban centres with increased social services for citizens. Typically priority actions for poverty alleviation and economic development include building sustainable livelihoods based on access to productive land and water, universal access to basic social services, improving entrepreneurial opportunities, credit and micro-financing schemes, technical and administrative training, and the development of appropriate technologies [1]. However, this development trajectory is contingent on maintaining healthy ecosystems that are capable of provisioning ecological services such as clean water, stable fertile agricultural soils and sources of revenue (such as through tourism) to the citizens.

How can the benefits of economic development be realized without compromising the natural resources and ecological integrity on which a country depends? We use a planned transportation corridor through northern Tanzania as a case-study to compare the relative benefits of three possible routes in terms of securing people’s basic needs (such as food, education, and healthcare) and infrastructural support (such as linking to markets, labour, and industry). We conduct a spatial socio-economic analysis on a broad set of data including the topography of the landscape, construction costs, travel times, transportation and haulage efficiency, agricultural spin-offs, and the advancement of education, employment, and health opportunities for the people in the Mara-Shinyanga region. Specifically we aim to find a solution that maximizes the socio-economic development of the Tanzanian people without compromising the ecology and exclusive tourism industry of the Serengeti, while simultaneously strengthening the trade routes that link the Indian Ocean to Lake Victoria.

### A case study: Tanzania and the Serengeti road

Tanzania is well placed in East Africa to become a major commercial hub of the region. The country has a long coastline and shares borders with 8 trading neighbours, 5 of which are completely landlocked. It has experienced 5% to 7% economic growth per annum in the last decade, yet despite this financial expansion there persists an alarming degree of human poverty [2]. Recent estimates indicate that as much as 74% of Tanzania’s 44.9 million people live below the poverty line [2–4]. Although economic projections predict that rural poverty will decline in the coming years assuming that Tanzania’s GDP continues to rise by 7% per annum, the reliability of these indicators is debatable [5]. Tanzania’s impressive growth forecasts are primarily due to increased mining and an expanding export market, however continued progress depends on (i) a favourable global economic environment, (ii) improved efficiency and accountability of public management and governance, (iii) structural reforms to improve the expansion of small and medium sized private enterprises, (iv) strengthened human capital through improvements to education, health and social welfare of the labour force, and (v) expanded and well-developed infrastructure such as transportation, electricity and service corridors [3, 6].

Building new infrastructure such as roads is integral for catalysing economic development in Tanzania [6, 7]. According to the Africa Infrastructure Country Diagnostic report by the World Bank, Tanzania requires an additional US$2.9 billion dedicated to development if it is to achieve par with more developed nations [4]. In particular, the transportation infrastructure in Tanzania is below the average of other sub-Saharan countries [8]. For instance, only 6.7% of the 103,706 km of roads in Tanzania are paved which poses a serious impediment to the country’s development [2]. Shortfalls in transportation capacity, electrical interruptions, and water supply account for at least a 34% reduction in Tanzania’s potential output [7]. Simulations suggest that improved infrastructure and roads would increase Tanzania’s per capita growth rate by as much as 3.4% [4].

The development of new roads and infrastructure requires careful planning so as not to jeopardize the country’s sources of foreign revenue from tourism. In 2010 Tanzania generated US$1.279 billion from tourism alone [2]. The Serengeti-Ngorongoro ecosystem is the world’s most famous protected area and is best known for the annual migration of 1.3 million wildebeest that support the highest density of predators anywhere in Africa [9]. Currently the revenue generated by tourism in the Serengeti-Ngorongoro ecosystem alone (including entrance fees for the park and buffering conservation areas, hunting licences, lodge fees, overnight fees) is in excess of US$100 million / year—a revenue source worth safeguarding. A preliminary assessment of a road through Serengeti aimed at a capacity of 3,000 vehicles and transport trucks / day (equivalent to 125 vehicles / hour) [10], which would seriously jeopardize the exclusive product Tanzania currently markets in the Serengeti (note, more conservative estimates suggest there would be up to 300 transit vehicles / day).

Until now there has been limited analysis of the socio-economic benefits a road would have for the people in the region (with one notable exception [11]), let alone an economic cost-benefit comparison of all the routes that might justify why a road through the Serengeti is better than the alternative options. There is a clear and urgent need for a transportation backbone through northern Tanzania, and given the benefits a road would have, the primary question remaining is: which route would provide the most economic gain for Tanzania while simultaneously improving the social welfare of its people without compromising the ecological integrity and ecosystem services provided by the Serengeti?

#### Three possible transportation routes

**Fig 1:**
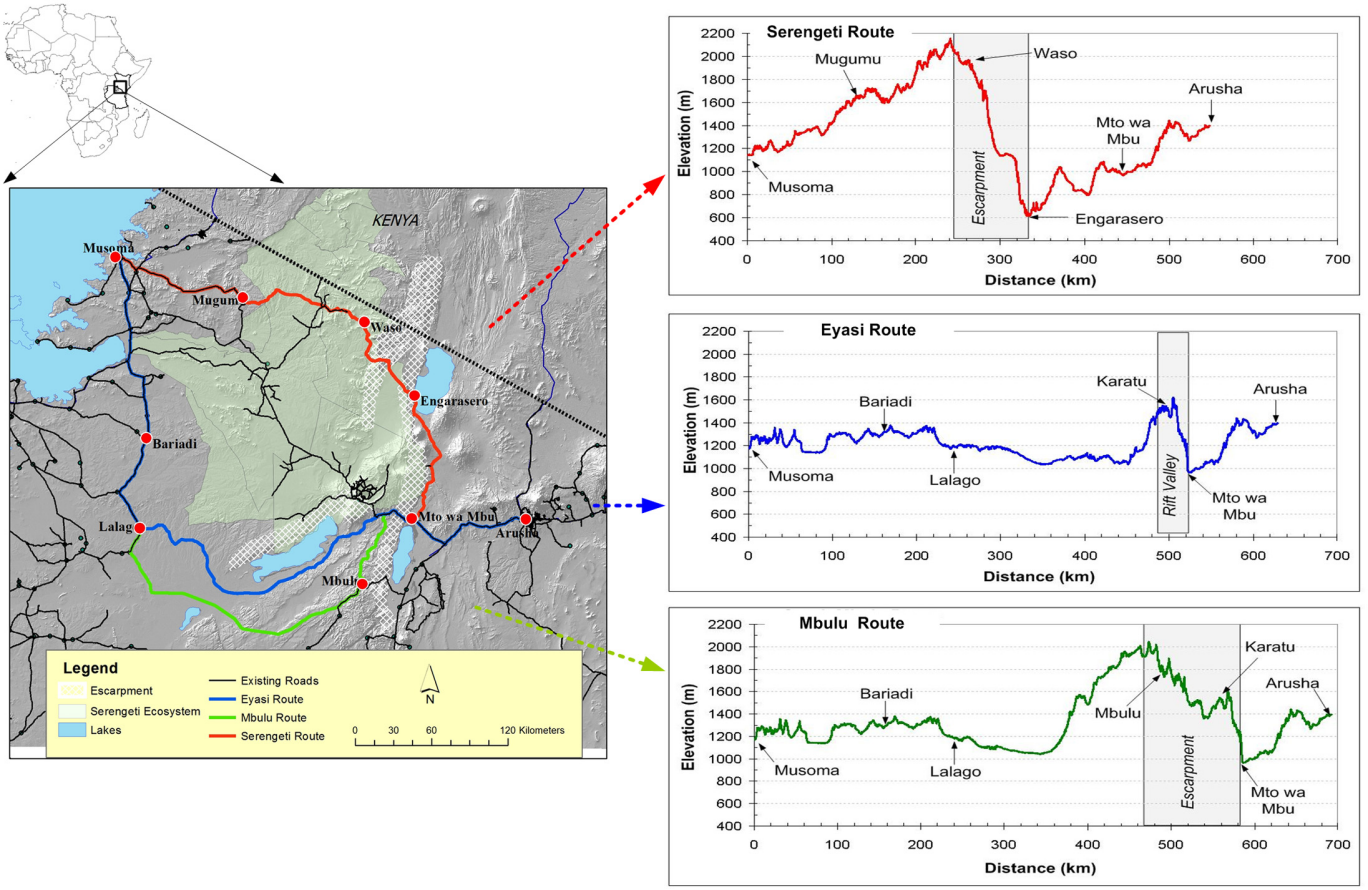
Three possible routes for a road connecting northern Tanzania to Lake Victoria. The Serengeti Route is 548km with an elevation gain of 1537m, the Eyasi Route is 628km and gains 674m, and the Mbulu Route is 692km and with an elevation change of 1099m. Results suggest the construction and haulage costs would be greatest for the Serengeti Route and least for the Mbulu Route (adapted from [50]).

Reports commissioned by the Government of Tanzania outline three potential routes to connect the regional road networks of Lake Victoria to Arusha [10, 12]. These routes (Fig 1) are the Serengeti Route that would follow the Rift Valley northward to Lake Natron, ascend the escarpment, transit through Loliondo and across the northern extension of the Serengeti National Park to Mugumu and then pass directly east to the ports in Musoma on edge of Lake Victoria. A second option would skirt south of the Ngorongoro Crater, around the bottom of Lake Eyasi to Lalago before travelling north to Musoma via Bariadi (the Eyasi Route). A third route, the Mbulu Route, is similar to the second but would extend further south connecting Mbulu to Lalago and Bariadi before returning northwards to Musoma. Information about the sources of the spatial data sets and details of the GIS analysis are outlined in S1 Appendix.

## Results and Discussion

Ideally a new road should stimulate the economy sufficiently such that over the long term not only are the initial costs of construction recuperated, but the road will eventually generate additional revenue for the country by increasing trade and thereby attracting international investment (Tanzania’s Medium-Term Public Investment Plan stipulates a 10% economic rate of return must be met before public funds can be committed) [13, 14]. In addition, a new road should be placed strategically so that this front-end investment raises the living standards of the most people and makes the largest contribution to poverty alleviation. Research indicates that even a limited amount of infrastructure development, such as access to a road, significantly improves livelihoods by increasing the level of education, health care and provisioning graduates with better employment opportunities than subsistence agriculture [15, 16]. In the following sections we systematically conduct a cost-benefit analysis weighing the socio-economic spin-offs associated with building each of the proposed roads, against the potential ecological impacts a road through the Serengeti might have.

### Comparison of distance, elevation and construction costs

In a first-pass analysis we used a digital elevation model (DEM) from NASA’s Shuttle Radar Topology Mission (SRTM) to describe each of the three proposed routes. From this we calculated the elevation profile of each (S1 Appendix) and compare the costs and potential barriers that could block the development of roads, railways and pipelines crossing the East African Rift Valley.

The results of the elevation profile indicate that the Serengeti Route has the greatest and steepest gradients while the Eyasi Route has the least (Fig 1). The elevation gain of 1537m over an 80km stretch along the Serengeti Route poses many potential problems. First, the gradient ascending the Rift Valley walls exceeds 10% in certain sections, which precludes the possibility of a viable railway associated with the road. Typically a locomotive is capable of pulling half its normal load on gradients of 1%; a 10% gradient would severely limit the total haulage capacity of this route. Second, oil and gas pipelines are prone to bursts and catastrophic leakages when the gradient is greater than 0.8% because of the pressure associated with sudden elevation changes [17]. The gradient of the Serengeti transit corridor could make this route logistically unviable for a pipeline that would potentially connect to the Ugandan gas reserves, whereas the Eyasi or Mbulu Route would be more feasible because the elevation is lower and the gradient is less. Third, the costs of carving switch-backs and building retaining walls for a road and rail system along the Serengeti Route would be far greater than either of the other two possible routes. Currently TanRoads, the government body responsible for the construction and maintenance of roads in Tanzania, estimates that a bitumen road of this standard would cost between 630,000 to 750,000 USD/km, however these estimates double and triple if support structures such as retaining walls and complex drainage ditches are required [18]. Fourth, the elevation gain of the Serengeti Route makes it the most costly for road freight. A 10 tonne truck would require approximately 15.1 litres of petrol to climb the Rift Valley along the Serengeti Route as compared to 6.6 litres along the Eyasi Route, and 10.8 litres along the Mbulu Route (or 14.1, 6.2 and 10.1 litres of diesel, respectively) (calculations are described in Table A in S1 Appendix). Therefore, the physical topography of the Serengeti Route makes it the most logistically challenging with the most expensive haulage costs.

### Comparison of road networks and travel times

Ideally the construction of a new road should link together as many other existing roads as possible to provide an efficient national transportation network that decreases the costs and uncertainty associated with land transport, rather than being an isolated project [7, 19]. The government of Tanzania is investing heavily in road construction as a means of building its infrastructure capital and spurring economic growth. Of the 52 current road construction projects in Tanzania, 18 are funded by the Government of Tanzania, 15 are financed by multilateral aid, 5 are financed by bilateral aid, 8 by the World Bank, and 6 are funded by the Millennium Challenge Corporation (www.tanroads.org). Chinese companies account for 72% of the awarded construction contracts, 9% of the road construction companies are from the East African Community, 11% are European, and the remaining 8% are companies from South Africa, South Korea, and Japan. If a new road through northern Tanzania is placed strategically, it could be part of a comprehensive interlinked transportation network connecting the Indian Ocean ports to Central Africa (i.e. the Central Development Corridor; S2 Appendix), or the trans-African highway linking the east and west coasts [20]. A simple node count of the major road intersections (i.e. the major trunk roads recognized by TanRoads) suggests that the Serengeti Route connects the fewest existing road networks in the region and would contribute the least to a national transportation network (7, 14, 16 major intersections for the Serengeti, Eyasi, and Mbulu routes respectively; Fig 1a; Table 1).

**Table 1:** A comparison of the total distance of each proposed route, the current surface conditions, the amount of new paving, the number of major junctions, and the total human population the road would access.

Proposed Route	Total Km	Km Currently Paved	Km Currently All-Seasonal Murum	Km Currently Seasonal Tracks or No Road	Km of new pavement required	Number of new junctions (node count)	Total human population within 10km
Serengeti Route	547.8	119.5	219.7	208.6	428.2	7	1,038,901
South Eyasi	628.3	296.0	83.1	249.33	332.4	14	1,687,359
South Mbulu	691.5	288.5	132.2	270.8	402.9	16	1,964,366

Note: The Serengeti Route requires the most amount of new pavement, would be the most costly to build, would contribute least to a national transportation network, and connects the fewest people.

The current status and road conditions of each of the 3 proposed routes was also assessed in order to estimate the total amount of new bitumen that would be required for each route (Table 1). Although the Serengeti Route is the shortest total distance (548km) it requires the most amount of new pavement (428km). The Eyasi Route (628 km total) requires 332 km of pavement and would connect to 296 km of existing paved road. The Mbulu Route is 63 km longer than the Eyasi Route (total is 691km) and would connect 288 km of existing road and would require 403 km of new pavement.

A major objective in building this road is to improve the transit of goods between the Lake Victoria region and the Indian Ocean, therefore we should not only consider the haulage costs of the three routes, but also the travel times. The speed limits in Tanzania are tiered by land zone. The maximum permitted speed for transport trucks is 80km/hr on all national highways however this drops to 50km/hr in protected areas. Table 2 compares the travel times based on the distance inside and outside protected areas for each of the three routes. Although the Serengeti Route has the shortest distance between Arusha and Musoma, there is effectively no difference in travel times between it and the Eyasi Route (7 hours 54 minutes as opposed to 7 hours 48 minutes, respectively). The travel time of the Mbulu Route would be about 45 minutes longer than either of the other two. The Serengeti National Park does not allow night driving, therefore traffic on the Serengeti Route would face an additional restriction of travelling only during the 12 hours of daylight.

**Table 2:** A comparison of travel times for the three routes based on the maximum allowable speed inside and outside protected areas indicates there is very little difference between the Serengeti Route and the Eyasi Route.

Proposed Route	Total Km inside Protected Areas (50 km/hr speed limit)	Total Km outside Protected Areas (80 km/hr speed limit)	Total Travel Time
Serengeti Route	151.1	396.6	7.9 hours
South Eyasi	0	628.3	7.8 hours
South Mbulu	0	691.5	8.6 hours

### Comparison of economic returns and taxation

The development of a new road could increase the country’s tax revenue by attracting investment, generating jobs, and increasing the flow of money [21]. The catalytic effect of a new road on local economies would be maximized if it (a) provided small and medium sized entrepreneurial opportunities to the most number of people, (b) connected the region’s natural resources such as gold mines and cash crops to the industrial centres, while (c) simultaneously bringing a large work force from the surrounding districts to the industrial centres [4, 22]. Approximately 70% of Tanzanians live in rural communities where the primary income is based on agriculture. However, non-agricultural earnings account for 40% of a family’s income in communities that are connected by roads, which spreads the risk of seasonal unemployment when crops cannot be cultivated, and provides opportunities for other family members (e.g. youths, elders, or mothers) to engage in income generating activities for the household [22, 23]. The development of diversified income and non-agricultural enterprises in rural communities such as small-scale industry and manufacturing are key to poverty alleviation [16] because they provide alternate sources of income and an economic cushion [23]. Almost 72% of Tanzania’s GDP in 2010 was based on these small and medium sized enterprises [2], however their establishment is constrained by a lack of transportation, electricity, and logistical support from financial institutions rather than a lack of financial capital itself [22]. A well-placed transportation corridor could improve the success of small and medium enterprises by developing supply chains, access to regional labour pools, and provide an export market, which could transform rural areas from a state of agricultural dependency to active economic growth and diversification [4, 16, 24]. Therefore, it is plausible that the least costly route may also have the fewest beneficial spin-offs and therefore be an economic or political “white elephant”.

As a means of assessing the potential economic return of a new road we analysed the demographic data from the 2002 Tanzanian census to determine which route connects the areas of greatest tax returns with the largest centres of unemployment. The data are collated from household interviews conducted by enumerators from the Tanzania Bureau of Statistics (S1 Appendix).

**Fig 2:**
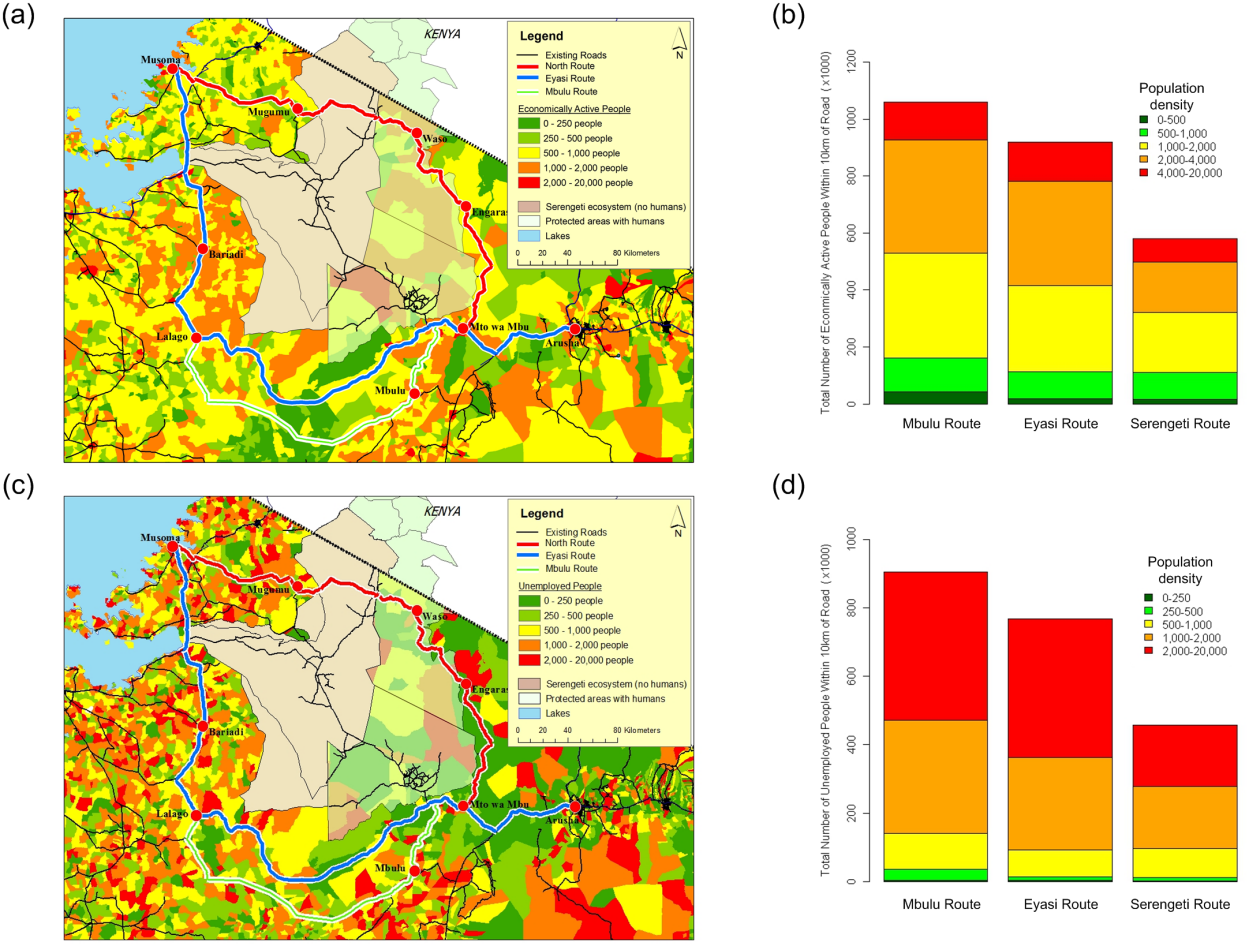
A comparison of the socio-economic demographics of the three possible routes. (a) The spatial distribution of economically active people and (b) the total number of economically active people within 10km of each route (Mbulu = 1,059,436; Eyasi = 919,297; Serengeti = 580,864); (c) the spatial distribution of unemployment and (d) the total number of unemployed people within 10km of each route (Mbulu = 904,930; Eyasi = 768,062; Serengeti = 458,037). The data suggest the Mbulu Route would connect the most unemployed people the largest centres of economic activity.

The results suggest the Mbulu Route followed by the Eyasi Route would have the greatest economic potential. Either of these routes would connect more than 900,000 economically active people located in several centres of high activity (Fig 2a and 2b) with an additional 700,000 unemployed people (Fig 2c and 2d) and would fuel further economic development in the region. By comparison, the Serengeti Route has approximately 55% of the economic activity of either the Mbulu or Eyasi routes as well as about half the number of unemployed people. The low economic returns of the Serengeti Route combined with the large initial costs required to build it, suggests that it would easily take Tanzania more than twice as long to recuperate the costs of building the Serengeti Route as it would for either of the two southern routes. This alone would turn off most international investors and raise alarms for the Tanzanian financial institutions that are intent on relieving their country’s debt burden [4].

### Comparison of current and future agricultural diversification

Currently agriculture accounts for about 28% of the nation’s GDP and supports the livelihood of about 80% of the population, however the sector has an exceptionally large growth potential [2]. The Tanzanian government is committed to expanding the agricultural sector as a means of alleviating rural poverty and boosting the economy through their Kilimo Kwanza policy (Agriculture First), which aims to avail new land and modernize agricultural techniques (including the provision of enhanced finance mechanisms, incentives for private investment, and access to regional markets) [25]. Estimates from 2008 indicate that as much of 34% of the population are undernourished (predominantly in the under-five age class), even though the country has the capacity to grow more than enough food for itself. It is possible that a new transit corridor could not only link existing agricultural centres with regional markets (thereby having an immediate effect), but also open new areas in which Tanzania’s agricultural sector could develop in the future [4]. Therefore, in addition to the immediate benefits gained by connecting existing agriculture we also investigate the long-term potential of the three routes to expand agriculture outside protected areas as this might potentially warrant putting a road where it would not otherwise be considered.

#### Current agriculture

**Fig 3:**
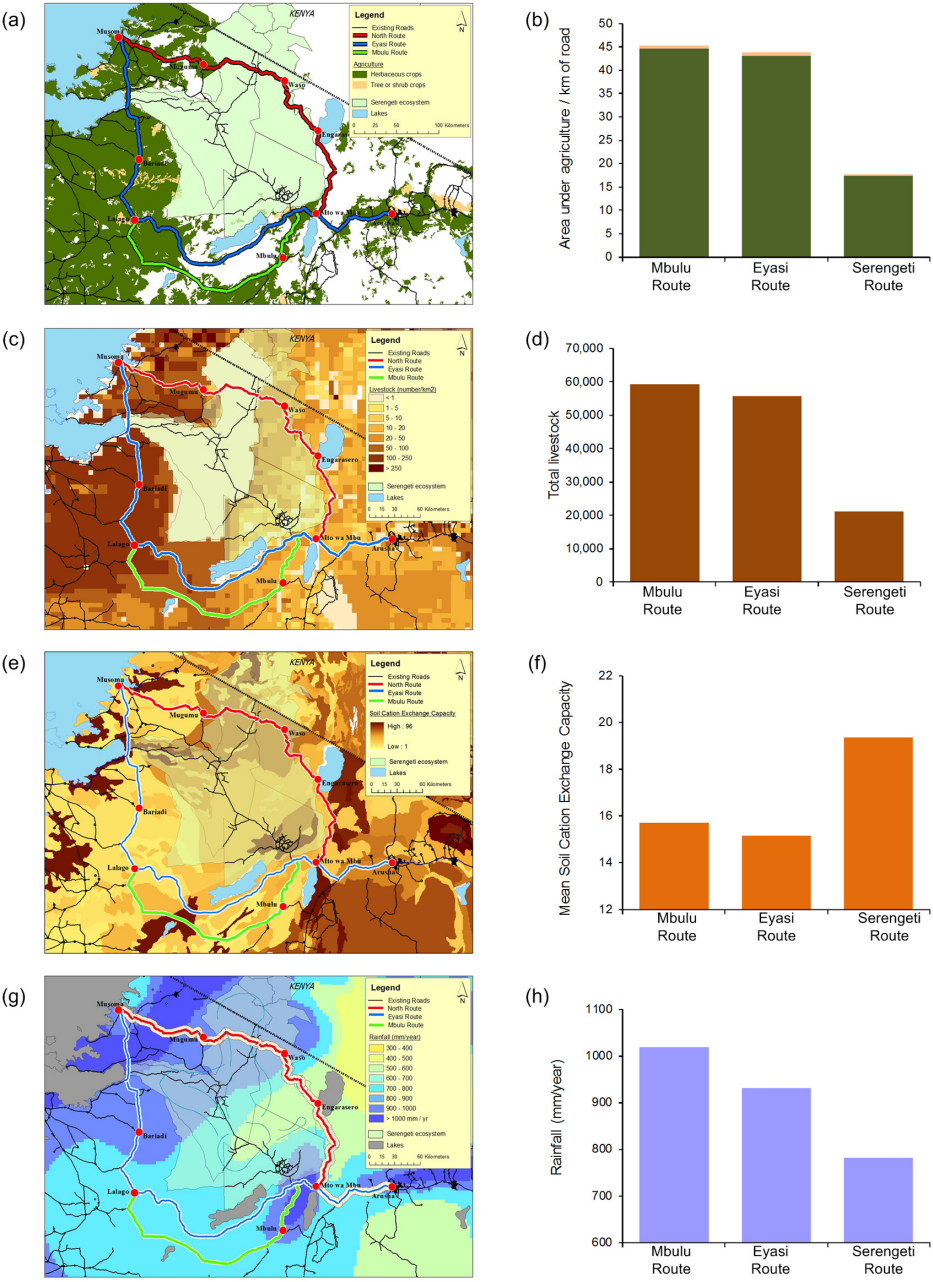
A comparison of the agricultural productivity of each route. (a) The spatial distribution of current crops (herbaceous crops are generally maize and beans for subsistence, shrub crops are generally cotton for cash), (b) the total amount of agriculture per kilometre of road; (c) the density of livestock and (d) the total livestock within 20km of each route. The potential for future agriculture depends on soil fertility and rainfall: (e) the distribution of soil fertility (estimated from the soil’s cation exchange capacity) and (f) the average soil fertility of each route; (g) the distribution of the mean annual rainfall and (h) the total annual rainfall along each route.

Ground-truthed satellite remote sensing data from the Food and Agriculture Organization (FAO; S1 Appendix) illustrate the majority of cultivation in this region tends to be annual herbaceous crops such as maize, beans, and cassava (Fig 3a)[26]. These are typical subsistence food items and are also sold in local and regional markets, but are rarely traded internationally. There are relatively few areas with perennial shrub crops, such as cotton, which are typically cash crops and depend on accessing large international markets. Of the three possible routes, Mbulu currently has the greatest amount of subsistence and cash crop agriculture (Fig 3b). A road from Mbulu to Lalago would provide best links between agricultural production centres and the regional markets in Arusha, Mwanza and Musoma. By comparison the Serengeti Route produces less than a third of the agriculture of the Mbulu Route and has no cash crops.

Livestock are the major source of protein for people during the dry season and are relied on heavily once the herbaceous crops have senesced; they are also a primary source of income to rural agriculturalists and pastoralists. We analysed livestock density data from the FAO (S1 Appendix) in relation to each of the three proposed routes (Fig 3c) [27]. The data suggest the Serengeti Route would access the least livestock, even though this area is dominated by Maasai pastoralists known for their animal husbandry skills. The Mbulu Route, however, connects the highest density of livestock to the greatest number of people thereby improving the access to regional markets by the most agriculturalists (Fig 3d). Consequently the Mbulu Route would have the combined effect of improving local economies for the most people, while also provisioning surrounding regions with the largest protein supply during periods of paucity.

#### Future agricultural potential

Approximately 1.7% of Tanzania’s land mass is currently under permanent agriculture, however surveys estimate that as much as 11.3% is arable [2]. This discrepancy between Tanzania’s current agricultural output and its potential output is largely driven by (a) inadequate access and provision of supplies to farmers and (b) few opportunities for them to sell their products in markets [4]. If the aims of improving and diversifying the agriculture sector are to be met (as per the Kilimo Kwanza policy), where should these efforts be focused? In this semi-arid region of Africa, any agriculture depends on fertile soils combined with adequate rainfall.

The potential for agricultural expansion was assessed by calculating the total mean annual rainfall and the cation exchange capacity of the soil (i.e. the soil’s ability to release the cations that are required by plants to grow) for the areas within 10km of each of the three routes (S1 Appendix). The rainfall data are from FAO and United Nations Environment Programme and the soil data from the Soil and Terrain Database for Southern Africa [28].

The analysis indicates that the soil fertility is greatest along the Serengeti Route (Fig 3e and 3f) due to volcanic ash deposits from Ol Donyo Lengai that frequently nourish the soils with elements such as sodium, potassium and magnesium. However, these areas also receive the least amount of rain (Fig 3g and 3h) so, although they are fertile, much of the region is too arid to support agriculture (i.e. below 600mm/year). Furthermore, many of the areas along the Serengeti Route have incompatible landuse practices such as pastoralism, hunting and ecotourism and are not zoned for agriculture [29]. The Mbulu Route has the greatest annual rainfall with moderately fertile soils, and therefore has the largest potential to support future agriculture making this route the most valuable prospect of the three, while a road through the Serengeti is the least viable option.

#### Pastoralism

If the intention of a new road is to alleviate the state of poverty in the Maasai pastoralists that live to the east of the Serengeti, these benefits are likely to be overshadowed by significant risks. For instance, the reduced travel time and improved access to pastoral areas are likely to attract immigrants looking to buy land with the intention of converting rangelands into croplands. As repeatedly witnessed in other Maasai regions across East Africa (e.g. Kajiado, Narok, and Monduli), the tendency to convert communal rangelands into fenced plots with mechanized agriculture leads to the fragmentation of seasonal livestock grazing areas [14] which ultimately displaces pastoralist people into dryer, lower production landscapes [30]. This loss of land leads to insecurity especially in poorer households that traditionally depend on livestock and grazing rights for income, food and even building materials [31]. Furthermore, as immigration into these traditional pastoralist areas increases, the local communities may struggle to maintain control over their natural resources such as grazing rights and therefore lose the ability to sustainably manage them [32]. Although poverty is widespread in the Maasai areas, there is one level of paucity below the current state, and that is Maasai without land. The competitive international trade that a highway would bring poses a distinct risk for the traditional lifestyle of Maasai people, especially if adequate landuse planning and laws are not already in place to protect them.

### Comparison of relative contributions to childhood education

Currently, 18.3% of the Tanzanian government’s annual expenditure is dedicated to education however the primary school student-teacher ratio still remains at 51:1 [2]. The challenges in educating children are most pronounced in rural areas [33] where the benefits of formal schooling tend to be undervalued by poor families because there is a perception the cost of educating children confers no advantages. However, studies show that connecting rural areas to regional labour markets provides channels for families to generate sufficient income to cover school fees as well as providing employment opportunities for graduates [15, 16]. This pattern partially accounts for the increased number of children now completing primary education in Tanzania (55% in 1991 to 90% in 2010 [2]), however opportunities for secondary and higher education are not common [33, 34]. The construction of a new road would provision the crucial infrastructural backbone that will eventually lead to mainline electricity, water, mobile phone communication and internet all of which significantly raises the level of education in rural areas [15]. If the Serengeti Route improved the provisioning of education to more school children above that of the alternative routes, this could justify building a road through the ecosystem.

**Fig 4:**
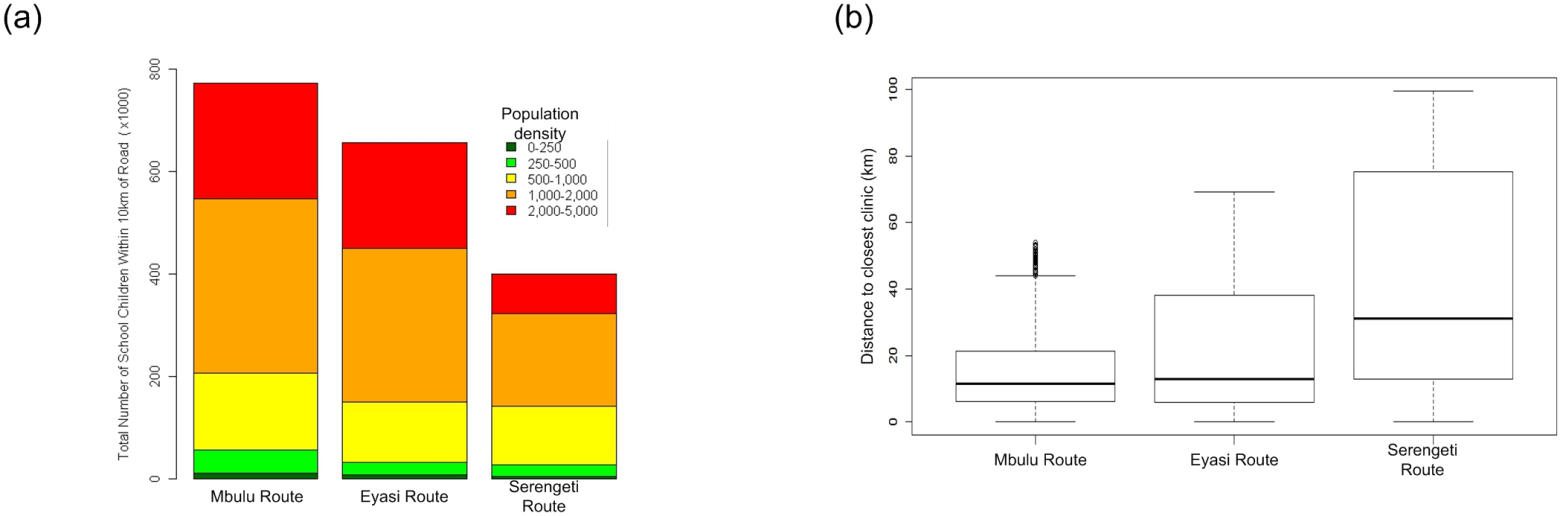
A comparison of the education and health care opportunities for the people living along each route. (a) The total number of school aged children within 10km of each route partitioned by density and (b) the mean distance (km) to the closest clinic for people living along each route. The results show the Serengeti route connects the fewest children to schools and is the poorest at connecting rural people to medical facilities.

We ask the question: which route would connect more school aged children to schools and therefore have the largest impact on educating future generations? Using the demographic data from the 2002 Tanzanian census, the total number of school aged children within 10 kilometres of each of the three possible routes were compared. The results (Fig 4a) show that the Mbulu Route connects twice as many school children as the Serengeti Route. In addition, the Mbulu Route has the most children living in low-income rural areas and would have the largest impact on improving rural education opportunities. The Serengeti Route would contribute the least to the education objectives of the country because it connects the fewest children to schools.

### Comparison of impacts to healthcare

The Government of Tanzania faces huge demands in improving the healthcare situation for its citizens. As much as 56% of rural Tanzanians cannot access suitable health care in less than one day of travelling, which remains a major challenge for the nation [2, 33]. Treatable diseases, maternal and prenatal complications, and poor nutritional condition account for 66% of all deaths [2] in Tanzania. Many of these deaths could be avoided if people had better access to facilities and if medical facilities were better equipped. For example, there are approximately 0.7 hospital beds and 0.2 nurses per 1,000 people [2] and about 22% of mothers receive no pre-natal care (maternal death rate hovers just over 4%). Therefore, Tanzania’s healthcare sector faces at least two major challenges: first, to improve the current medical facilities including the number of doctors, nurses and midwives; and second to ensure fast and reliable access to clinics especially in rural areas [35].

What is the average distance to the nearest hospital for each route? A strategically placed road would have the combined effect of connecting isolated hospitals, medical supplies and expertise to other centres, as well as improving the access to medical facilities for local people. We compare the average distance to the closest clinic for each of the three routes. Because there is no geospatial location data for all the hospitals and clinics in the area, we assume that all towns have at least one clinic according to the mandate of the Tanzanian Ministry of Health [35]. The location of all towns was determined from the Google Map Maker project and the distance to each was calculated (S1 Appendix).

The average distance for any person living along the Mbulu Route to a hospital is 16.2 km (maximum is 53.8km), while the Eyasi Route is 21.4km (69.3km maximum) and the Serengeti Route is 41.5km (99.6km maximum) (Fig 4b). When this result is considered relative to the total number of people living along each route, the Mbulu Route would improve the quality of health and prenatal care to the most people and have the largest immediate impact on Tanzania’s medical sector (i.e. it would bring 1.96 million people to within 16 km of a medical facility on average). Conversely, a road through the Serengeti would connect 1.04 million people but would be compromised because there are no medical facilities inside the core protected area.

### Ecological impacts

The Serengeti is best one of the best studied ecosystems in the world and is known for the annual migration of 1.38 million wildebeest and 250,000 zebra [36] which move in a seasonal pattern between Tanzania and Kenya. The mass migration of animals drives virtually every other ecological component in the ecosystem, from the diversity of plants and insects, to the extent of the forests, to the predator-prey interactions [9, 37–40]. In addition, the ecosystem provisions water, grazing, soil nutrients as well as a vibrant tourism industry for the nation [41–44].

The Serengeti Route would bisect the migration and cut the ecosystem in half. In addition to transiting through the core range of a threatened population of black rhino and the endangered wild dog, this route would fracture the migratory path of the wildebeest and zebra. A recent simulation experiment showed that by separating the wet season calving grounds from the dry season refuge a road would could reduce the wildebeest population by 30% [45, 46]. A reduction of this magnitude would permanently change the dynamics of this ecosystem [36], and concurs with other studies investigating the impacts of roads in many other protected areas [47–49]. Furthermore, the synergistic effects of a declining wildebeest population and increased competition for agricultural land would have large negative impacts especially on the poorest people in the region, many of whom depend on the ecosystem for key resources [11]. Evidence suggests that the knock-on effects of a degraded ecosystem in terms of reduced tourism could reduce foreign revenue, which could alter the national exchange rate and ripple through the rest of Tanzania’s economy [11].

## Conclusions and Future Direction

The World Bank estimates that Tanzania could save almost a US$500 million a year through institutional reforms and infrastructure development; for instance, every dollar spent on roads and infrastructure generates US$4 for the Tanzanian economy [4]. Considering the population of northern Tanzania and the potential economic gain of connecting agricultural communities to regional centres of trade, it is clear that the country urgently needs a transportation corridor to improve the development of the region. This transportation corridor could become the back-bone that would eventually support electricity lines, high-speed fiber optical cables, water, and gas pipelines in addition to a road and rail service. Furthermore, a road connecting the Indian Ocean ports to Lake Victoria (and possibly on towards Rwanda, Burundi, and Uganda) would be hugely beneficial for the development of these landlocked states and would provision Tanzania with a remunerative transportation industry. However, care must be taken so that the economic benefits of a road do not compromise Tanzania’s natural heritage and tourism industry that generates US$1.279 billion per annum and depends on the conservation of intact pristine biodiverse areas.

Our independent analysis of the costs and benefits of three possible transportation routes across northern Tanzania clearly illustrates that the Serengeti Route is the most expensive with the least economic gain for the nation and risks the largest negative impact on conservation and tourism (Table 3). This route requires the most amount of new pavement, has the greatest elevation gain making road and rail transportation hugely inefficient, and has an equivalent travel time as slightly longer routes to the south of the Serengeti. Conversely, the Eyasi Route and the Mbulu Route access twice as many people as the Serengeti Route making either of these routes a politically savvy project for accessing the electorate. The Mbulu Route connects the most number of unemployed people to economic hubs and would join the largest labour force to the most entrepreneurial businesses. In addition, the Mbulu Route accesses the best agricultural areas, has the largest potential for future agricultural developments and links several regional supply chains which would improve food security in the region. Furthermore, the Mbulu Route also connects the most schools and hospitals and makes the largest contributions towards improving the social welfare of Tanzania’s people. The risks associated with the Serengeti Route in terms of threatening the integrity of the ecosystem, degrading its tourism value, and the large costs and poor returns means it has the least long-term benefits for Tanzania [11].

**Table 3:** A summary of the costs and benefits each of the three potential routes would provide to the economic and social development of northern Tanzania.

		Mbulu Route	Eyasi Route	Serengeti Route
Construction & Haulage Costs	Distance	*Worst*	Mid	**Best**
	Elevation	Mid	**Best**	*Worst*
	Fuel Consumption	Mid	**Best**	*Worst*
	Amount of New Pavement	Mid	**Best**	*Worst*
Development of New Infrastructure to Boost the Economy	Links to Existing Road Networks	**Best**	Mid	*Worst*
	Travel Times	*Worst*	**Best**	Mid
	Linking Hubs of Economic Activity	**Best**	Mid	*Worst*
	New Employment Opportunities	**Best**	Mid	*Worst*
	Tax Revenue	**Best**	Mid	*Worst*
	Linking Cash Crops	**Best**	Mid	*Worst*
Provision of Basic Needs to the Most People	Linking Subsistence Agriculture	**Best**	Mid	*Worst*
	Future Agricultural Potential (i.e. soil quality & rainfall)	**Best**	Mid	*Worst*
	Livestock	**Best**	Mid	*Worst*
	Protection of Pastoral Lifestyle	**Best**	Mid	*Worst*
	Education	**Best**	Mid	*Worst*
	Healthcare	**Best**	Mid	*Worst*

Note: The results suggest the Mbulu Route is most likely to meet the 10% economic rate of return threshold that is required for the investment of public funds while the Serengeti Route is the worst option.

If national development is to achieve long-term sustainability it must mitigate the ecological risks. For instance, the development of new infrastructure must provision for people’s basic requirements, spur the economic growth of industries and expand export markets, while simultaneously maintaining the country’s ecological integrity. It is only through detailed and comparative analyses that viable economic solutions can be achieved that do not compromise the health of the ecosystems on which future generations will depend.

## Supporting Information

S1 Appendix Data sources and methodology behind the spatial analysis including summary calculations estimating the total fuel required to cross the Rift Valley for each of the three routes (Table A). (DOCX)

S2 Appendix The central development corridor and Tanzanian policy. (DOCX)
