# Supplementary material for: Balancing Conservation with National Development: A Socio-Economic Case Study of the Alternatives to the Serengeti Road
Source: PLoS One. 2015 Jul 22;10(7):e0130577. doi: 10.1371/journal.pone.0130577 (PMC4511738; doi:10.1371/journal.pone.0130577)
Supplement: S1 Appendix — (DOCX) [file pone.0130577.s001.docx]

Supplementary Online Material

# S1 Appendix A: Data sources and methodology behind the spatial analysis

All the datasets used in this analysis are from freely available material that has been made accessible by development agencies and national governments as a means of increasing public engagement and improving sustainable development in the region. The data are suitable for a first-pass analysis to compare the spatial distribution of broad-scale socioeconomic returns between the three alternative transportation routes. The fine scale socioeconomic patterns relating to the degree of education, health care, and agricultural economies along each route can only be determined by on-the-ground field work which is currently underway, thanks to the support of the Kreditanstalt für Wiederaufbau Bank (KfW), the Intergovernmental Platform on Biodiversity and Ecosystem Services (IPBES), and the European Commission’s Framework 7 Program. However, the data from these field based research programs will take years to collate and given the speed at which the Government of Tanzania is developing strategies for this region, it is prudent to use the existing datasets to answer some of the most pressing questions immediately.

## Geographical and elevation data

A digital elevation model (DEM) was made in ArcGIS (ESRI, 2011) using version 2 of NASA’s Shuttle Radar Topology Mission (SRTM V2) data downloaded from the US Geological Survey’s EROS Data Center (<http://srtm.usgs.gov/index.php>). The SRTM V2 data provides accurate elevation information for the East African region at a resolution of 90m. Using the 3D Analyst tool in ArcGIS, the elevation profile of each route was calculated from the DEM and plotted from Musoma to Arusha. The maximum elevation gain from the lowest point to the highest point was calculated as well as the maximum rate of inclination in order to estimate the construction costs and the haulage capacities for each route.

## Fuel consumption calculations

The amount of fuel required to climb the Rift Valley was calculated as follows:

Potential energy required to get to top of Rift Valley (J) = Mass of loaded lorry (kg) * Elevation gain across Rift Valley (m) * Gravitational force (m/s2) * Coefficient of friction for dry tyres

Joules of energy required to climb Rift Valley for internal combustion engine (J) = Potential energy required to get to top of Rift Valley (J) / Maximum energy conversion efficiency for internal combustion engine

Litres of petrol (l) = Joules of energy required to climb Rift Valley for internal combustion engine (J) / Joules per litre of petrol (MJ/litre)

Table A. Summary calculations estimating the total fuel required to cross the Rift Valley for each of the three routes.

|  | **Serengeti** | **Eyasi** | **Mbulu** |
| --- | --- | --- | --- |
| Mass of loaded lorry (kg) | 10000 | 10000 | 10000 |
| Elevation gain across Rift Valley (m) | 1537 | 674 | 1099 |
| Gravitational force (m/s2) | 9.8 | 9.8 | 9.8 |
| Coefficient of friction for dry tyres | 1.7 | 1.7 | 1.7 |
| Potential energy required to get to top of Rift Valley (J) | 256064200 | 112288400 | 183093400 |
| Maximum energy conversion efficiency for internal combustion engine | 0.5 | 0.5 | 0.5 |
| Joules of energy required to climb Rift Valley for internal combustion engine (J) | 512128400 | 224576800 | 366186800 |
| Joules per litre of petrol (MJ/litre) | 34 | 34 | 34 |
| Joules per litre of diesel (MJ/litre) | 36.4 | 36.4 | 36.4 |
| Litres of petrol (l) | 15.0626 | 6.6052 | 10.7702 |
| Litres of diesel (l) | 14.06946154 | 6.169692308 | 10.06007692 |

## Existing road networks

Data on the existing road networks in the region came from 2 different sources. Roads and infrastructure within the Serengeti National Park were from GPS tracklogs collected by the National Parks GIS department (Roads_V5 in [www.serengetidata.org](http://www.serengetidata.org)). The spatial data of roads and towns outside the protected area boundaries were from Google Map Maker Project. Google’s Map Maker is an open source project through which users add and update geographic information about their region which is then shared via Google’s Map and Earth platforms. The information is checked and further updated by other users resulting in detailed accurate maps of the region which are freely accessible to the public.

## Socioeconomic and demographic data of Tanzania

The Tanzanian National Bureau of Statistics conducts a census every decade in which they attempt to enumerate every household in the country. During this nation-wide survey enumerators also collect information about gender and age of the occupants as well as the number of people employed (including self-employment). The processed data from the national census can be viewed from the National Bureau of Statistics website (<http://www.nbs.go.tz/>), however the raw data must be requested in person directly from their offices. Using the data from the 2002 census we calculated the number of eligible voters, the number of school aged children, the mean unemployment rates, the mean dependency rates, and the density (people/km^2^) for every district in the region. From this, we conducted a spatially restricted calculation in ArcGIS to estimate the total amount of economic activity, total number of people unemployed, and the total number of school children within 10km of each of the proposed routes. The data were partitioned by density enabling us to estimate not only how many people were present along each route, but also how the population was regionally divided. For instance, we were interested in differentiating routes that had centres of dense economic activity that were interspersed with areas of high unemployment from routes that had low economic activity with few centres of economic activity. The route with the highest potential for alleviating rural poverty should be one that connects the most labourers to the largest industrial centres and the most children to schools.

The access to medical facilities was calculated by creating a distance raster in ArcGIS from each major town in the region. The distance to the closest major town was measured every kilometre along each route and the means, quantiles and variance were plotted as box-and-whisker graphs for comparative purposes.

## Agriculture, livestock, rainfall, and soils

The spatial data on cultivation was accessed from the United Nation’s Food and Agriculture Organization’s (FAO) AfriCover website. AfriCover collects and collates time series geographical information pertaining to land use, climatic conditions, and natural resources. We used the Tanzania cultivation and agriculture layer which differentiates areas based on herbaceous crops such as maize, beans, and cassava from and tree/shrub crops such as cotton, coffee and tea. The data was published in 2002 and comes from LANDSAT TM satellite imagery that has been classed by the spectral reflectance of ground-truthed agricultural crops. The data is freely accessible via the website: <http://www.glcn.org/activities/africover_en.jsp> . In ArcGIS we determined the total area within 10km of each road that was under herbaceous versus shrub cultivation to assess the extent of subsistence based agriculture (i.e. herbaceous crops) as opposed to cash based agriculture (i.e. shrubs).

Data on the density of livestock in the region was from FAO’s Animal Production and Health department. The department’s mandate is to continually collect global livestock statistics at a sub-national level. The data are collated from national statistics and surveys and are interpolated where needed based on empirical relationships with environmental variables in similar agro-ecological zones. We calculated the total number of livestock based on the estimated density of animals within 20km of each route (i.e. the distance animals can be walked to and from markets). Our intention was to identify the routes that provide access to the most livestock and facilitate the local economies associated with agricultural husbandry. The livestock data are freely available at: <http://www.fao.org/ag/againfo/resources/en/glw/GLW_dens.html> .

The potential for developing new agriculture fundamentally depends on accessing areas of sufficient soil fertility with adequate rainfall. We used the Soil and Terrain Database for Southern Africa (SOTERSAF) to map the soil fertility in the region. The SOTERSAF database was developed by the International Soil Reference and Information Centre (ISRIC) which is an independent science-based foundation with a mandate of providing global soil data and education material in order to facilitate solutions to development issues. The data are collated from national archives and sample banks that have been collected over several decades. Using the SOTER database we determined the mean cation exchange capacity of the soils within 10km of each route to determine the potential for cultivation. The SOTER data is open access and can be downloaded from: <http://www.isric.org/data/soil-and-terrain-database-southern-africa-version-10-sotersaf>. In addition we used rainfall data compiled by the United Nations Environment Program to determine the mean annual precipitation within 10km of each route. UNEP’s rainfall database is part of a desertification risk assessment being conducted in collaboration with FAO. The raw data come from field measures and maps from national meteorological agencies which have been processed into a gridded rainfall map for the African continent available here: <http://www.grid.unep.ch/data/data.php>.

## References for S1 Appendix A

**ESRI** 2011 ArcGIS Desktop 10. Release 10 ed. Environmental Systems Research Institute, Redlands CA.
