# Supplementary material for: Balancing Conservation with National Development: A Socio-Economic Case Study of the Alternatives to the Serengeti Road
Source: PLoS One. 2015 Jul 22;10(7):e0130577. doi: 10.1371/journal.pone.0130577 (PMC4511738; doi:10.1371/journal.pone.0130577)
Supplement: S2 Appendix — (DOCX) [file pone.0130577.s002.docx]

Supplementary Online Material

# S2 Appendix B: The Central Development Corridor and Tanzanian policy

Tanzania has invested a considerable amount of planning into a transit and infrastructure backbone that would connect the gold mines in the north-east and the nickel and iron-ore deposits on the edge of Rwanda and Burundi to the Indian Ocean ports. This project, called the Central Development Corridor, has been welcomed by the East African Community because it would also facilitate the economies of the landlocked nations of Central Africa by provisioning alternative access to ports and international markets. Additionally, as international investors look towards the mineral rich regions of Central Africa, the motivation to finalize the planning and start construction the Central Development Corridor is mounting from both the perspective of the buyers and sellers.

The multilateral Central Corridor Transit Transport Facilitation Agency (<http://www.eastafricancorridors.org>) was established in 2006 by the governments of Tanzania, Kenya, Uganda, Rwanda, Burundi and the Democratic Republic of Congo to facilitate trade in the region by harmonizing national transportation policies. One of the many objectives of the agency is to ensure the laws and procedures relating to national infrastructure development for any one of the partner countries are not violated. For instance, according to national law the Government of Tanzania must meet several criteria before being able to authorize spending on national infrastructure projects. The Medium-Term Public Investment Plan (United Republic of Tanzania, 2009, Tanzania National Resource Forum, 2011) outlines six conditions that must be met before any public money can be invested, which includes an exhaustive environmental and social impact assessment authorized by the National Environmental Management Council (NEMC) and an economic feasibility study. Most notably the investment of public funds into infrastructure development projects must clearly and unequivocally demonstrate that a minimum Economic Rate of Return of 10% will be met. Therefore, a new road in Tanzania must not only comply with social and environmental standards, but it must also be economically viable (Tanzania National Resource Forum, 2011).

## References for S2 Appendix B

**Tanzania National Resource Forum** 2011 The Serengeti highway controversy; A layman's guide. *Swara* April-June Issue 14-21.

**United Republic of Tanzania** 2009 Guideline for the preparation of medium term plan and budget framework for 2009/10 –2011/12 Ministry of Finance and Economic Affairs, Government of the United Republic of Tanzania, Dodoma.
